# Development of a new humanized mouse model to study acute inflammatory arthritis

**DOI:** 10.1186/1479-5876-10-190

**Published:** 2012-09-13

**Authors:** Alexander V Misharin, G Kenneth Haines, Shawn Rose, Angelical K Gierut, Richard S Hotchkiss, Harris Perlman

**Affiliations:** 1 Department of Medicine/Rheumatology, Northwestern University, Feinberg School of Medicine, 240 East Huron Street, Room Chicago, IL 60611, USA; 2 Department of Pathology, Yale University, School of Medicine, New Haven, CT 06510, USA; 3 Department of Anesthesiology, Washington University School of Medicine, St Louis, MO 63110, USA

**Keywords:** Humanized mouse, Leukocytes, Rheumatoid arthritis, Etanercept

## Abstract

**Background:**

Substantial advances have been generated in understanding the pathogenesis of rheumatoid arthritis (RA). Current murine models of RA-like disease have provided great insights into the molecular mechanism of inflammatory arthritis due to the use of genetically deficient or transgenic mice. However, these studies are limited by differences that exist between human and murine immune systems. Thus, the development of an animal model that utilizes human immune cells, will afford the opportunity to study their function in the initiation and propagation of inflammatory arthritis.

**Methods:**

One to two-day old irradiated NOD-*scid IL2rγ*^*null*^ (NSG) mice were reconstituted with human CD34+ cord blood stem cells. Leukocytes were analyzed by flow cytometry and circulating antibodies were determined by ELISA. Arthritis was induced by injecting complete Freund’s adjuvant into knee or ankle joints. Mice were also treated with the TNF inhibitor, Etanercept, or PBS and joints were analyzed histologically.

**Results:**

Humanized mice were established with high reconstitution rates and were able to spontaneously produce human immunoglobulins as well as specific IgG in response to immunization. Intraperitoneal injection of thioglycolate or injection of complete Freund’s adjuvant into joints resulted in migration of human immune cells to the injected sites. Arthritic humanized mice treated with Etanercept had markedly less inflammation, which was associated with decreased total numbers of human CD45+ cells, including human lymphocytes and neutrophils.

**Conclusions:**

The humanized mouse model is a new model to study inflammatory arthritis disease using human leukocytes without rejection of engrafted tissue. Future studies may adapt this system to incorporate RA patient cord blood and develop a chimeric animal model of inflammatory arthritis using genetically predisposed immune cells.

## Background

Rheumatoid arthritis (RA) is a chronic autoimmune disease, which affects many organs and systems, but mainly attacks synovial joints and may lead to cartilage destruction and deformation, resulting in chronic pain, severe disability and increased mortality. Despite recent progress, our understanding of the etiology and pathophysiology of RA is still far from complete. Currently, there are numerous animal models of RA-like disease, including K/BxN arthritis, collagen-induced arthritis, antigen-induced arthritis, SKG mutant, *lpr*, and IL-1Ra^−/−^ mice [1], each of which mirror various aspects of RA. However, a common weakness of all models is the reliance on an entirely murine-based immune response to inflammation. As such, there have been instances where these models have led to contradictory results in therapeutic efficacy studies as compared to RA patients. For example, previous studies that examined the effect of biologic therapy in murine systems of RA-like disease have noted that inhibiting IL-1 provided suppression or complete amelioration of arthritis. However, anti-IL-1 therapy, although very successful in treating autoinflammatory syndromes and systemic juvenile idiopathic arthritis [2], has limited efficacy in RA patients. One of the closest murine models to human RA is the transgenic mouse expressing the human HLA-DRβ1*0401 (DR4) gene, which develops an RA-like disease following stimulation with collagen or citrullinated peptides [3]. While this model represents a tremendous discovery as the initiation of the disease is mediated by the expression of the share epitope, it still retains the limitations of all the previous models of RA-like disease, namely that the inflammation is driven by the murine immune system that ectopically express the shared epitope. The central reason for discrepancies between animal models and patients may be attributed to differences between human and murine immune systems. These differences affect both innate and adaptive immunity, including the balance of leukocyte subsets, defensins, toll-like receptors (TLR), inducible NO synthase, NK inhibitory receptor families Ly49 and KIR, FcR, Ig subsets, B cell (BLNK, Btk, and λ5) and T cell (ZAP70 and common γ-chain) signaling pathway components, Thy-1, γδT cells, cytokines and cytokine receptors, Th1/Th2 differentiation, costimulatory molecule expression and function, antigen presenting function of endothelial cells, and chemokine and chemokine receptor expression [1]. Thus, development of an inflammatory arthritis model using human cells would be useful to understand how the human immune system responds during the course of inflammatory arthritis, and may direct the development of future therapies with improved efficacy as well as selectivity.

Until recently, many attempts to engraft human immune cells into various immunodeficient mice resulted in poor and short-term engraftment, which was mainly attributed to residual activity of the host’s immune system. To overcome these issues three major improvements were made over the last 15 years. First, was the generation of SCID (*Prkdc*^*Scid*^) mice, which have a mutation in the protein kinase, DNA-activated, catalytic polypeptide (*Prkdc*) gene and thus lack T- or B-cells but still have high NK-cell activity. These mice were able to sustain a limited and transient engraftment of the human immune system. A second improvement was the introduction of the *SCID* mutation into the NOD background, which has substantially decreased activity of NK-cells, deficiency in C5, and inability of macrophages to produce IL-1β in response to stimulation with LPS, as well as other defects of the innate immune system [4]. Both SCID and NOD-SCID mice have been widely used to study the engraftment of synovial tissue from RA patients [5]. One study has even shown short-term reconstitution of human bone marrow stem cells in an arthritis model [6]. However, these models either do not have a functional human immune system and/or support long-term engraftment of hematopoietic cells. A third generation of immunodeficient mice have the deletion of the *IL2rγ* gene which is also known as the common cytokine-receptor γ-chain, and is required for IL-2, IL-4, IL-7, IL-9, IL-15 and IL-21 signaling, and its absence significantly affects functioning of the innate immune system (such as monocytes and neutrophils) and completely prevents NK-cell development [4]. The three immunodeficient mouse strains that employ this advantage are: BALB/c-*Rag2*^*−/−*^*IL2rγ*^*−/−*^, NOD-*Rag1*^*−/−*^*IL2rγ*^*−/−*^, and NOD-*scid IL2rγ*^*null*^ (the latter is commonly referred as NSG for **N**OD **s**cid **g**amma). The inactivation of the gamma chain of the IL-2 receptor has dramatically improved the engraftment of human cells. While the only cell types that remain in these immunodeficient mice are neutrophils, monocytes/macrophages, and dendritic cells, they are hypofunctional [7], which is evident by the lack the inflammatory immune response to bacterial and fungal pathogens [8]. These characteristics allow not only transfer of human peripheral blood mononuclear cells (PBMC), but also support long-term engraftment of human hematopoietic stem cells (HSC). Over time, engrafted HSC undergo multilineage development, resulting in a fully functional human immune system, including T, B, NK and dendritic cells, as well as monocytes/macrophages and granulocytes. Human T cells undergo positive and negative selection in the thymus (which prevents development of the graft versus host disease), display a diverse repertoire of T cell receptors, exhibit human leukocyte dependent cytotoxicity, and produce a delayed type hypersensitivity response. Mature B-cells expressing functional B-cell receptors are readily detected as well as circulating IgM and IgG. Macrophage and dendritic cell production of cytokines and chemokines and presentation of antigens to T-cells have all been demonstrated in the humanized mouse [4,9]. This humanized mouse model helped the progression of studies on human-specific infectious diseases, such as HIV, Dengue virus and *Salmonella typhi* for which animals are not susceptible [4,9]. Moreover, it also uncovered pathophysiological mechanisms involved in sepsis in humans [10]. However, the tremendous potential of this model to study human autoimmunity has been minimally explored. Here, we developed a unique humanized mouse model for acute inflammatory arthritis. The major strength of this model is the ability to compare and contrast the activity of human immune cells prior to and during the course of inflamma-tory arthritis, which cannot readily be accomplished in patients.

## Methods

### Mice

NOD-*scid IL2rγ*^*null*^ (NSG) mice (Jackson Laboratory) were maintained at the barrier and specific pathogen-free facility at the Center for Comparative Medicine at Northwestern University. The Institutional Animal Care and Use and IRB committees (STU00024421) at Northwestern University approved all procedures. Humanized mice were generated using a modified protocol as described [11]. Twelve to twenty-four hour NSG pups were irradiated with 100 cGy and then reconstituted with 0.5x10^5^ human CD34+ hematopoietic stem cells (HSC) (Lonza, NJ) via intrahepatic injection. Alternatively, irradiated newborn pups were injected with 1x10^5^ CD34+ cells isolated from peripheral blood of healthy adult volunteers using CD34+ microbeads (Miltenyi, CA). Peripheral blood from humanized mice was obtained and analyzed using flow cytometry at the indicated time points. Reconstitution was considered to be successful if the number of human CD45+ cells in peripheral blood was more than 10% by week 10. Peritonitis was induced using aged 4% thioglycollate broth.

### Flow cytometry

Peripheral blood, spleen, bone marrow, and peritoneal cavity were processed as previously described [12]. After Live/Dead staining with Aqua dye (Invitrogen, CA) single cell suspensions (~1x10^6^ cells) were stained with a mixture of fluorochrome-conjugated antibodies against human CD45 (HI30), human CD4 (RPA-T4), human CD8 (RPA-T8), human CD14 (M5E2), human CD15 (HI98), human CD19 (HIB19), human CD56 (B159), human CD68 (eBioY1/82A), mouse CD45 (30-F11), mouse CD11b (M1/70), and mouse Gr-1 (RB6-8C5) antibodies. For intracellular staining (human CD68), cells were fixed in 2% paraformaldehyde and permeabilized in 0.1% saponin. Samples were acquired on a BD LSR II flow cytometer (BD Biosciences) and compensation and analyses were performed using FlowJo software (Treestar Inc, OR).

### B cell responses

To evaluate the specific adaptive B cell response of the human immune system in humanized mice, three 12-week old humanized mice were challenged with 3 IP injections of 2 μg of ActHIB vaccine (Aventis Pasteur, France), performed at 3- and 4- week intervals, respectively. Levels of HIB-specific antibodies were determined by ELISA (DEMEDITEC Diagnostics Gmbh, Germany). Immunoglobulin production in a cohort of 5 humanized mice was assessed using MILLIPLEX MAP Human Immunoglobulin Isotyping Kits (Millipore, MA).

### Adjuvant-induced arthritis in humanized mice

To induce arthritis in humanized mice, 10 μL of complete Freund’s adjuvant (CFA) 4 mg/mL (Chondrex, Redmond, WA) or vehicle (incomplete Freund’s adjuvant) were injected into the knee joint or into the periarticular space around the ankle. Additionally mice were treated subcutaneously with Etanercept (Enbrel, Amgen, Thousand Oaks, CA, and Pfizer Inc.) at 0.8 mg/kg on day −2 and day 0 or with PBS. Anti-Gr-1 antibody (0.25 mg) was administered (IP) on days −1 and 1 to deplete mouse neutrophils. Arthritis and ankle joint processing was performed as previously described [12]. Percent staining, bone damage, and inflammation scores were assessed by a pathologist (G.K.H.) blinded to the study [12-17]. Tibio-talar and knee joints of humanized mice were scored separately for bone erosion, inflammation, pannus formation and infiltration with specific cell subsets. The following criteria were used for inflammation: 0 – normal; 1 – minimal infiltration of inflammatory cells in periarticular tissue; 2 – mild infiltration; 3 – moderate infiltration, with moderate edema; 4 – marked infiltration, with marked edema; and 5 – severe infiltration, with severe edema. Pannus formation was scored according to the following criteria: 0 – normal; 1 – few pannus formation at 1 to 2 position; 2 – moderate pannus formation at 1 to 2 position; 3 – pronounced pannus formation with accretion to cartilage; 4 – pronounced pannus formation with cartilage overgrow; 5 – pronounced cartilage destruction due to pannus invasion. Bone erosion was scored according to the following criteria: 0 – normal; 1 – Small areas of resorption, not readily apparent on low magnification, in trabecular or cortical bone; 2 – More numerous areas of resorption, not readily apparent on low magnification, in trabecular or cortical bone; 3 – Obvious resorption of trabecular and cortical bone, without full thickness defects in the cortex; loss of some trabeculae; lesions apparent on low magnification; 4 – Full thickness defects in the cortical bone and marked trabecular bone loss, without distortion of the profile of the remaining cortical surface; 5 – Full thickness defects in the cortical bone and marked trabecular bone loss, with distortion of the profile of the remaining cortical surface.

### Statistics

Comparison between experimental and control groups was performed using Student’s unpaired two-tailed t-tests or, when appropriate, a Mann–Whitney test.

## Results

### Generation and characterization of humanized mice

**Figure 1:**
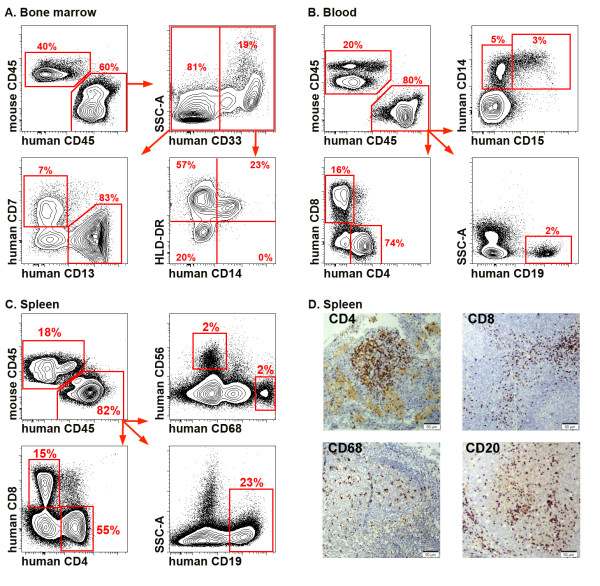
**Multilineage development of the human immune system in humanized mice.** Samples were prepared for flow cytometry as described in the Materials and Methods. Dead cells and debris were excluded on FSC-A vs. SSC-A dot plot, doublets were excluded on FSC-A vs. FSC-H dot plots (not shown). (**A**) Multilineage development in bone marrow of a 12-week old humanized mouse. Both myeloid and lymphoid lineages were present. (**B**) Peripheral blood of a 12-week old “humanized” mouse. Mature forms of B cells (CD19+), CD4 and CD8 T cells, monocytes (CD14+) and neutrophils (CD15) can be easily identified. (**C**) Spleen of a 12-week old “humanized” mouse: CD4+ and CD8+ T cells, CD19 B cells, macrophages (CD19-CD68+) and NK cells (CD56+) are shown. (**D**) Immunohistochemical staining for human T cell markers, CD4 and CD8, B cell marker, CD20, and macrophage marker, CD68, in the spleen of 12-week old humanized mouse. Magnification 20x. Data are representative of >20 mice.

**Figure 2:**
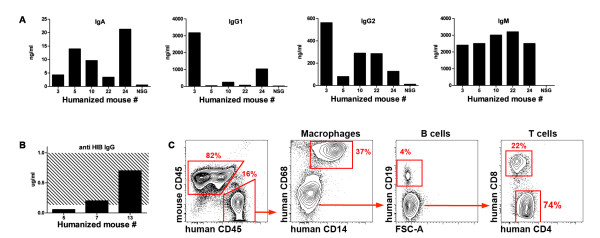
**Characterization of the humanized mouse.** (**A**) Spontaneous production of immunoglobulins by 16-week old “humanized” mice. Serum from a non-reconstituted NSG mouse was used as a negative control. (**B**) Production of the anti-HIB immunoglobulin 4 weeks after the third immunization with Act-HIB. (**C**) Human immune cells were found in the peritoneal cavity 72 hours after intraperitoneal injection of thioglycollate. All mature cell types, including T and B cells and macrophages were present.

Other groups have previously utilized NSG mice to examine infectious disease models, but their usefulness for the study of autoimmune diseases, such as RA, is unknown. Thus, we applied the NSG mouse model to the study of inflammatory arthritis. One-day old irradiated NSG pups were reconstituted with human CD34+ cord blood stem cells, similar to prior studies [11]. Engraftment was documented using flow cytometry and immunohistochemistry. Multilineage development of human leukocytes in primary and secondary lymphoid organs was detected (Figure 1A-D). The percent engraftment (40-60%) of the human immune system, their stages of development, and the survival of reconstituted humanized mice were comparable to previous reports [18,19]. Humanized mice spontaneously produced human immunoglobulins (Figure 2A) as well as specific IgG in response to immunization with ActHIB vaccine (Figure 2B). In contrast to cord blood stem cells, CD34+ stem cells isolated from peripheral blood of healthy adult volunteers were unable to reconstitute the immune system of irradiated newborn NSG mice (data not shown), consistent with a recent report [19]. Innate immune system function in humanized NSG mice was also investigated using a sterile peritonitis thioglycollate injection model. Human macrophages and neutrophils were readily detected in the elicited peritoneal cavity (Figure 2C). Taken together and consistent with previous studies [19-21], these data indicate that both innate and adaptive immunity are functional in humanized mice.

### Treatment with the TNF inhibitor Etanercept decreases the severity of CFA-induced arthritis

**Figure 3:**
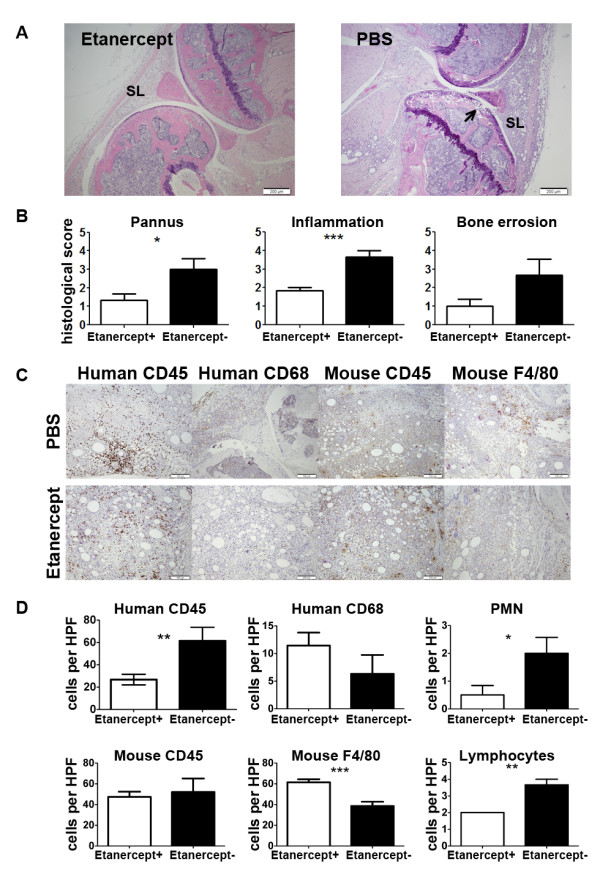
**Histopathological changes in joints of humanized mice 7 days after the initiation of the CFA-induced arthritis. **(**A**) H&E staining showing CFA-induced infiltration of the soft tissues of humanized mice treated with Etanercept (left) and PBS (right), magnification 20x. SL = synovial lining. Arrow denotes invasion site. (**B**) Histopathological scores for pannus formation, inflammation, and bone erosion in CFA-treated mice. (**C**) Immunohistochemistry for human CD45, human CD68, mouse CD45, and mouse F4/80 in humanized mice treated with PBS (upper row) and Etanercept (lower row). Magnification 40x. (**D**) Immunohistopathological scores for infiltration of human CD45+ cells, human CD68+ cells, mouse CD45+ cells, mouse F4/80+ cells, neutrophils, and lymphocytes in humanized mice treated with PBS or Etanercept. Data represents 5 (Etanercept) and 3 (PBS) mice/group.

A well-established model of inflammatory arthritis in mice is the adjuvant-induced arthritis model [22]. The applicability for this model as a representative system for human cells in the joint was examined. Injection of complete Freund’s adjuvant (CFA) resulted in the development of clinical and histological arthritis, as evidenced by the presence of swelling, erythema, decreased function, infiltration of immune cells, and signs of bone erosion (Figure 3A). In contrast, injection of incomplete Freund’s adjuvant resulted in much less ankle swelling, erythema and infiltration (data not shown). Since progenitors of murine neutrophils are radio-resistant, and to eliminate the possibility that murine neutrophils were contributing to the development of arthritis in NSG mice injected with CFA, anti-Gr-1 antibodies were administered to reconstituted humanized mice. Pretreatment with anti-Gr-1 antibody eliminated virtually all-murine neutrophils in blood (Additional file 1: Figure S1) and tissues (data not shown), but had no effect on the development of arthritis. This finding suggests that human cells were responsible for the development of arthritis in these animals.

To investigate whether the humanized mouse model might be suitable for testing therapeutics effective in RA, reconstituted humanized mice were treated with the TNF inhibitor Etanercept prior to the initiation of CFA-induced arthritis. TNF inhibition was chosen, as this agent is the most common and effective biologic therapy for RA patients. Humanized mice were randomly assigned to treatment (Etanercept) or control (PBS) groups. Groups did not differ in terms of age, sex or percentage of human CD45+ cells in peripheral blood. Treatment with Etanercept decreased histological scores for pannus formation by 2.3-fold (p = 0.0306), inflammation by 2.0 fold (p = 0.0008), and bone erosion by 2.7-fold (p = 0.071) as compared to PBS-treated mice (Figure 3B). The number of CD45+ human cells was also reduced by 2.3-fold (p = 0.0037) in the joints of Etanercept-treated mice, compared to control arthritic mice (Figure 3C, D). In contrast, there was no difference in the number of mouse CD45+ cells in the joint between the groups (Figure 3C, D). Further, the infiltration by human neutrophils and lymphocytes was reduced by 4.0-fold (p = 0.048) and 1.8-fold (p = 0.009) respectively in Etanercept-treated mice, compared to control arthritic mice (Figure 3C, D). Although not significant, Etanercept-treated animals had slightly increased number of human CD68-positive cells ( p = 0.2238) and but had significantly increased number of mouse F4/80-positive cells (61.5 vs. 38.56, p = 0.001). Our data demonstrate that in this murine model, human cells cause erosive, inflammatory arthritis that can then be ameliorated by a TNF-α inhibitor, which is the first-line biologic therapy for RA. Thus, we have proof-of-concept that this model may be used to study the activities of human immune cells in inflammatory arthritis, at predetermined time points, and in response to various therapeutics. Such controlled scrutiny of the human immune response to inflammatory arthritis has not been feasible until now.

## Discussion

Over the past several years, multiple editorials and reviews on the current state of immunology research have now suggested a need to refocus on human, and not mouse, immunology [1,23,24]. Some fundamental differences between murine and human immune systems impede the translation of basic science findings to the bedside. For example, multiple genes and proteins that are present in humans do not have direct homologs in mice and vice versa [25]. Indeed, recent comparison of gene expression profiles between human and mouse monocytes, which are critical in the pathogenesis of RA, revealed significant differences, including CD36, CD9, CXCR4, TREM-1, IL-1, NOS, arginase, and PPARγ [1,24]. These studies showed strikingly different patterns of receptors involved in the uptake of phagocytic cargo, and production of reactive oxygen species and IL-1 between human and mouse monocyte subsets. Additionally, other differences between human and mouse immune systems include the ratio of leukocyte subsets, expression of Toll-like receptors 2, 3, 9 and 10, as well as signaling components in B and T cells [1,25,26]. Thus, the differences between human and mouse leukocyte activity in response to inflammation may slow the advancement of research in the field of autoimmunity. Overcoming these obstacles will accelerate the pace of novel discoveries in disease pathogenesis and therapeutic targets. Notably, one model that has been suggested to be reminiscent of human RA is the human HLA-DR4 transgenic mouse. In this model all murine antigen-presenting cells express the human HLA DR4 gene and this allows for presentation of human epitopes such as citrullinated peptides, which leads to inflammatory arthritis development. This model is a great step forward for understanding the role that the shared epitope plays in the development of RA-like disease. Nonetheless, the antigen presenting cells, the T-cells, and all leukocytes originate from the mouse, which function differently than humans. Future studies may combine the HLA-DR4 transgenic mouse model and the humanize mouse model to bring together the strengths of both systems for studying RA-like disease. A recent study accomplished a similar task and crossed the NODShi-scidIL-2γR−/− (NOG) mice with mice that ectopically express human HLA gene to understand lymphocyte immune responses [27].

Development of the systemic model of rheumatoid arthritis in humanized mouse is the ultimate goal [28]. This ideal model would utilize hematopoietic cells derived from patients with rheumatoid arthritis and should be driven by clinically relevant immune response (i.e. to collagen or citrullinated peptides). However, this would have to occur without using peripheral blood stem cells as we have observed that these stem cells are not sufficient to reconstitute the immune system. Thus, other sources of stem cells such as induced pluripotent stem cells or cord blood stem cells from patients who have RA as the offspring may carry similar genetic predispositions for RA. While currently available NSG mouse provides much better engraftment and development of the human immune system than previous models (SCID or NOD-SCID mice), the functionality of the human immune system in humanized mouse is still limited. In particular development of B cells and production of antibodies in response to immunization are impaired [29]. Deficiency of complement C5 component, which NSG mice have due to their NOD background, leads to decreased activity of the residual mouse immune system [8], however, on the other hand, it may decrease response of the human immune system as well. Recently, improved immunodeficient NSG mouse strains were generated and support better development of myeloid and lymphoid cells [30-34], allowing for development of the humanized mouse model that may closely reproduce rheumatoid arthritis. While these strains are not widely available we have chosen local injection of the CFA as a simple and robust and way to assess usefulness of the currently available humanized mouse model to test anti-arthritis therapy.

Here, we have generated a humanized mouse by transferring isolated cord blood stem cells into irradiated one-day old NSG mice. These mice develop a fully functional human immune system that is capable of innate and adaptive immune responses. Furthermore, the human leukocytes are able to traffic and extravasate into primary and secondary lymphoid organs and to inflamed tissues. While other animal models of RA have examined the role of IL-1, we chose to focus on TNF-α inhibition, as anti-IL-1 therapy has overall inferior clinical efficacy compared to TNF-α inhibitors in RA patients. Experimental inflammatory arthritis develops in this mouse and is suppressed by a TNF-α inhibitor and is associated with reduction of human but not mouse leukocytes in the joint (Figure 3). However, there was no effect on macrophage numbers, which may be due to the time of harvesting the joints or that these macrophage have an altered phenotype and become responsible for the resolution phase of the disease. Future studies will be required to further understand the role that the human macrophages play in this model. Interestingly, previous studies have shown that anti-IL-1 therapy is successful at ameliorating AIA [35,36], while anti-TNF therapy has yielded conflicting results in the AIA model [36-39]. Since TNF inhibition is considered to be the first-line biologic therapy, the fact that human cells respond better than the murine cells indicates that the humanized mouse system has the potential to replicate the responses that occur in patients. While the acute inflammatory arthritis that results from injecting adjuvant into the joints of mice does not entirely reflect the complexities of the adaptive and innate immune responses that occur in RA, additional studies using other models of RA-like disease will provide more information on the suitability of this new model. Further, future studies will aim at recreating a spontaneous arthritis using stem cells from RA patients carrying HLA alleles strongly associated with the high risk of RA. However, the fact that we have shown recruitment of engrafted human cells, their physiologic response to adjuvant in the form of inflammatory arthritis, and reduction of disease burden with TNF-α inhibition that directly correlates with decrease in numbers from the joint, suggests the feasibility of performing such experiments using genetically susceptible samples from patients in the future.

The humanized mouse has already had a profound impact on infectious disease research including HIV and vaccine development. Only recently have a few studies utilized the potential of the humanized mouse to understand the pathogenesis of autoimmune disease, such as type 1 diabetes [40]. Brehm and colleagues showed that human islet cells were capable of restoring normal glycemic levels in NSG-Akita mice and that human leukocytes derived from stem cells were capable of rejecting human islet allographs [41]. Further, injection of peripheral blood mononuclear cells from SLE patients into Balb*Rag2*^*−/−*^*IL2rγ*^*null*^ mice led to autoantibody production and increased mortality as compared to injection of cells from control patients [42]. More recently, Kuwana and colleagues reported spontaneous development of erosive arthritis in humanized mice infected with Epstein-Barr virus [43], which was mostly T cell driven with very few macrophages and B cells involved in the joint pathology. These data together with our findings suggest that the humanized mouse model may provide a suitable system to validate novel therapeutics for autoimmune disease.

## Conclusions

The goal of this manuscript is to provide proof-of-concept that human immune cells in the mouse system will behave in a manner that is reminiscent of humans. The first step toward developing a model that is similar to human RA is to demonstrate that human immune cells in a model of inflammatory arthritis behave in a manner that mirrors patients. Herein we have determined that the humanized mouse model of acute inflammatory arthritis permits the trafficking of human leukocytes to the joint, perhaps using mouse adhesion receptors on the endothelial surface [44], and produce pro-inflammatory molecules while in the joint. Moreover, treatment with the first-line biologic used in RA patients ameliorates arthritis and correlates with a reduction in human leukocytes but not mouse leukocytes in the joint. Thus, we propose a novel, humanized mouse model to dissect the intricacies of human immune cell activities in the initiation, propagation, and resolution of inflammatory arthritis.

## Abbreviations

AIA, Adjuvant-induced arthritis; CFA, Complete Freund’s adjuvant; HSC, Hematopoietic stem cells; IP, Intraperitoneal; NSG, NOD-*scid IL2rγ*^*null*^; PBS, Phosphate buffered saline; RA, Rheumatoid arthritis; TLR, Toll-like receptor.

## Competing interests

The authors declare that they have no competing interests.

## Authors’ contribution

AVM helped with study design, performed experiments, analyzed and interpreted data, and wrote the manuscript, SR performed experiments and analyzed data, GKH helped with study design and, analyzed and interpreted data, RH helped with study design and, analyzed and interpreted data, and contributed to the writing of the manuscript, AG helped with study design and, analyzed and interpreted data, and contributed to the writing of the manuscript and HP helped with study design, analyzed and interpreted data, and wrote the manuscript. All authors read and approved the final manuscript.

## Supplementary Material

Additional file 1 **Figure S1.** Depletion of murine neutrophils with anti-Gr-1 antibody. Peripheral blood from humanized mouse was collected before and 24 hours after IP injection of 0.25 mg of anti-Gr-1 antibody (clone RB6-8C5) and analyzed by flow cytometry. After gating out debris and doublets, human and mouse cells were identified as positive for human CD45 and mouse CD45 correspondingly. Mouse neutrophils (N) were identified as CD11b+Gr-1^hi^SSC^hi^ and monocytes (M) as CD11b+Gr-1^int/low^SSC^low^. Numbers on the contour plots indicate percentage of the parent population. Click here for file
