# Supplementary figures and images for: Development of a new humanized mouse model to study acute inflammatory arthritis
Source: J Transl Med. 2012 Sep 13;10:190. doi: 10.1186/1479-5876-10-190 (PMC3480927; doi:10.1186/1479-5876-10-190)

## Slide 1
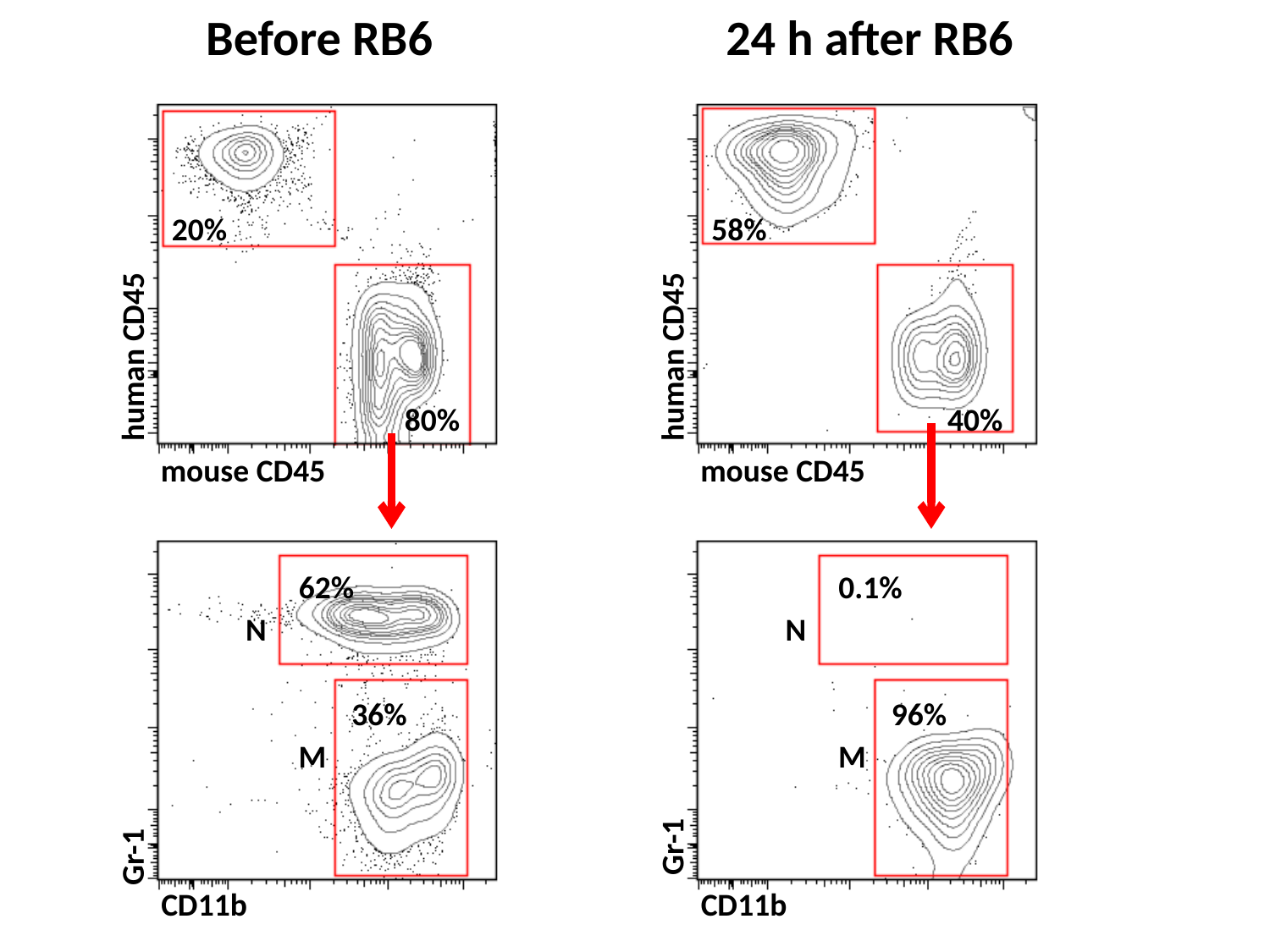

Before RB6
24 h after RB6
20%
58%
human CD45
human CD45
80%
40%
mouse CD45
mouse CD45
62%
0.1%
N
N
36%
96%
M
M
Gr-1
Gr-1
CD11b
CD11b

Supplement: Additional file 1 — Figure S1. Depletion of murine neutrophils with anti-Gr-1 antibody. Peripheral blood from humanized mouse was collected before and 24 hours after IP injection of 0.25 mg of anti-Gr-1 antibody (clone RB6-8C5) and analyzed by flow cytometry. After gating out debris and doublets, human and mouse cells were identified as positive for human CD45 and mouse CD45 correspondingly. Mouse neutrophils (N) were identified as CD11b+Gr-1hiSSChi and monocytes (M) as CD11b+Gr-1int/lowSSClow. Numbers on the contour plots indicate percentage of the parent population. [file 1479-5876-10-190-S1.pptx]
